# Response of the mosquito immune system and symbiotic bacteria to pathogen infection

**DOI:** 10.1186/s13071-024-06161-4

**Published:** 2024-02-17

**Authors:** Manjin Li, Yang Zhou, Jin Cheng, Yiqing Wang, Cejie Lan, Yuan Shen

**Affiliations:** 1 The Affiliated Wuxi Center for Disease Control and Prevention of Nanjing Medical University, Wuxi Center for Disease Control and Prevention, Wuxi, 214023 China; 2 Nanjing Medical University, Nanjing, 211166 China

**Keywords:** Mosquito, Pathogen infection, Innate immune system, Immune priming, Symbiotic bacteria

## Abstract

Mosquitoes are the deadliest animal in the word, transmitting a variety of insect-borne infectious diseases, such as malaria, dengue fever, yellow fever, and Zika, causing more deaths than any other vector-borne pathogen. Moreover, in the absence of effective drugs and vaccines to prevent and treat insect-borne diseases, mosquito control is particularly important as the primary measure. In recent decades, due to the gradual increase in mosquito resistance, increasing attention has fallen on the mechanisms and effects associated with pathogen infection. This review provides an overview of mosquito innate immune mechanisms in terms of physical and physiological barriers, pattern recognition receptors, signalling pathways, and cellular and humoral immunity, as well as the antipathogenic effects of mosquito symbiotic bacteria. This review contributes to an in-depth understanding of the interaction process between mosquitoes and pathogens and provides a theoretical basis for biological defence strategies against mosquito-borne infectious diseases.

**Graphical Abstract:**

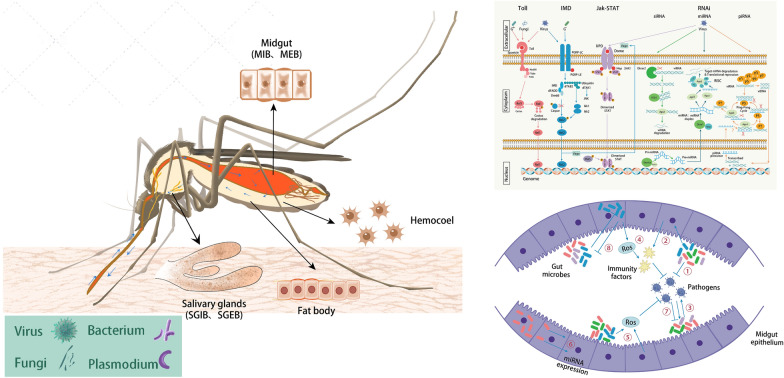

## Background

As an important pathogen vector, mosquitoes transmit a variety of insect-borne infectious diseases, such as malaria, dengue fever, yellow fever, and Zika, causing more deaths than any other vector-borne pathogen [1]. As the world's number one “health killer”, mosquitoes pose a great threat to global public health. Pathogens enter the mosquito body mainly in the following two ways: bacteria and fungi easily enter the body through brakes in the cuticle or exoskeleton; viruses, *Plasmodium*, and other parasites easily enter the midgut through mosquitoes sucking blood that contains the pathogens and penetrate the midgut tissue barrier through haemolymph diffusion to reach other organs and tissues, such as the trachea, fat body, and salivary glands, which allows them to cross the salivary gland barrier, enter the saliva, and transmit to the next host through bites.

Infection with arboviruses induces an innate immune response in mosquitos that activates a complex mosquito-pathogen interaction, which in turn alters the gene expression profile of mosquitos. Similar to other arthropods [2], mosquitoes lack an acquired immune system and rely primarily on innate immunity to protect against viruses, bacteria, fungi, parasites, and other pathogens [3, 4]. The mosquito innate immune system modulates infection by human pathogens or resistance to insect pathogens and increases the fitness and longevity of infected mosquitoes [1]. According to studies, cell-mediated phagocytosis, melanisation, and lysis are the three main modes of pathogen clearance by mosquitoes [5]. Cellular and humoral immunity are involved in the regulation of pathogen infection in mosquitoes, first through the recognition and binding of pathogens by pattern recognition receptors (PRRs) and pathogen-associated molecular patterns (PAMPs) and then through the activation of immune-associated signalling pathways, thus triggering cellular and humoral immunity in mosquitoes. Cellular immunity defends against pathogen invasion through the circulation of insect peripheral haemolymph, with phagocytosis and encapsulation responses mediated by haemocytes and pericardial cells. Humoral immunity, which also occurs in the haemocoel, involves a variety of mechanisms, including the production of antimicrobial peptides (AMPs), the activation of melanization by the phenoloxidase (PO) cascade, and the production of reactive oxygen species (ROS) and nitric oxide (NO) [1, 3]. There is no clear boundary between the molecular pathways of action of cellular and humoral immunity, and some of the molecules that participate in humoral immunity are produced by haemocytes and participate in cellular immunity at the same time [6]. Although mosquitoes only have innate immunity, there is some evidence that they can induce an enhanced immunity when they encounter reinfection, called “immune priming” (also known as trained immunity). In addition, the immunological role of mosquito symbiotic bacteria (including gut microorganisms and *Wolbachia*) will be further investigated. This review provides an overview of mosquito innate immune mechanisms in terms of physical and physiological barriers, pattern recognition receptors, signalling pathways, cellular and humoral immunity, and immune priming, as well as the antipathogenic effects of mosquito symbiotic bacteria.

## Physical and physiological barriers

**Fig. 1:**
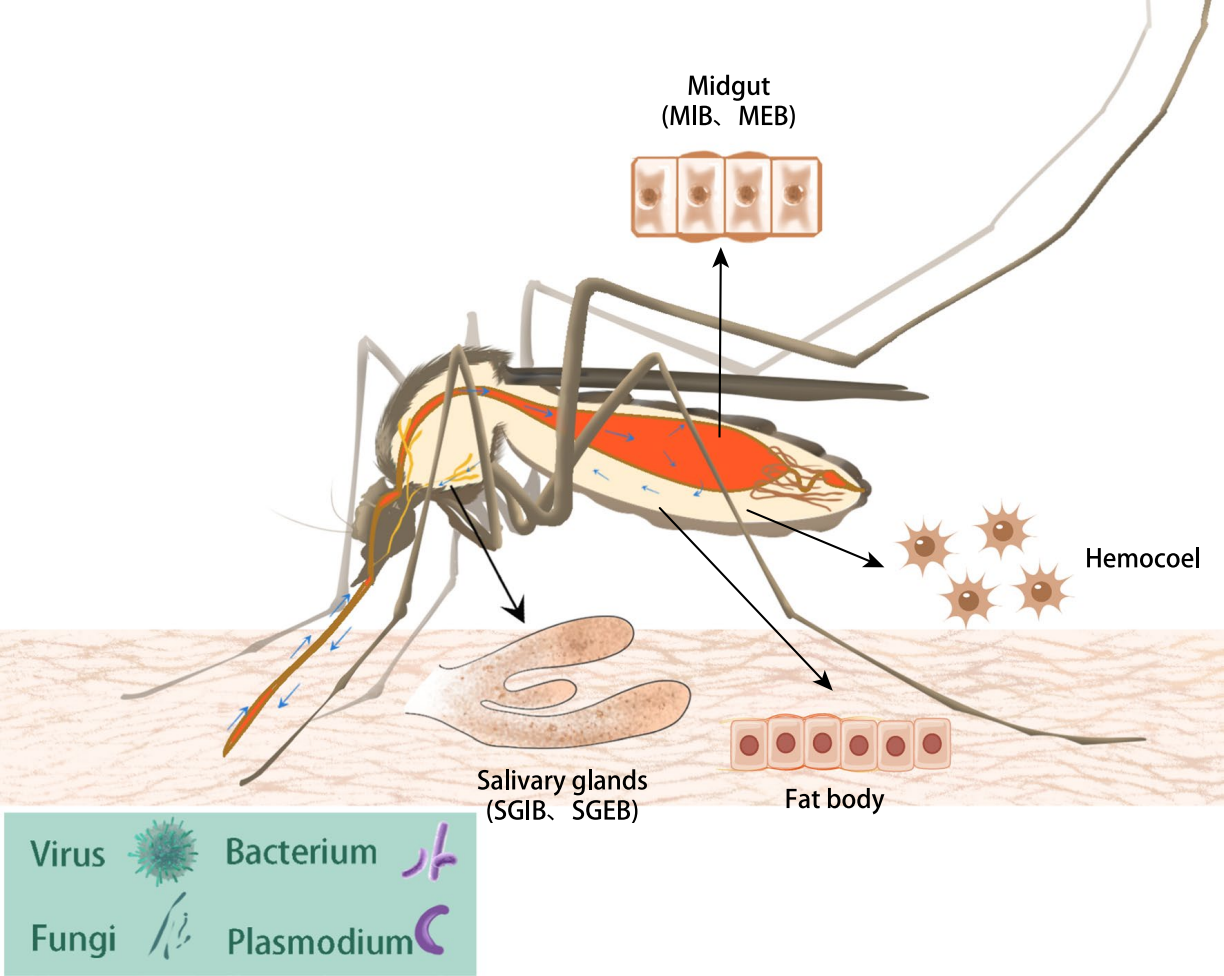
Main immune-related compartments of mosquitoes. The small blue arrows indicate the general circulation pattern of the pathogens in mosquito after the mosquito feeds on blood infected with the pathogen. Pathogens include viruses, bacteria, fungi, *Plasmodium*, etc. The pathogen enters the infected blood, partly develops in the intestinal epithelium, partly excretes from the intestines, and is passively transported through the mosquito's circulatory system as it crosses the haemocoel to reach the salivary glands. The large black arrows indicate the four main immune compartments, including the midgut [represented by midgut epithelial cells; containing the midgut infection barrier (MIB) and the midgut escape barrier (MEB)], salivary glands [containing the salivary gland infection barrier (SGIB) and the salivary gland escape barrier (SGEB)], haemocoel (represented by haemocytes), and fat body (represented by trophocytes and oenocytes)

The physical barriers of mosquitoes mainly include the exoskeleton, cuticle, epithelial-epidermal barrier, trachea, fat body, gut, haemocoel (including haemolymph and haemocytes), and salivary glands. Four of these compartments, the midgut, haemocoel, salivary glands, and fat body, play dual roles as important physical and physiological barriers (Fig. 1). There are four main physiological barriers: midgut infection barrier (MIB), midgut escape barrier (MEB), salivary gland infection barrier (SGIB), and salivary gland escape barrier (SGEB). Physiological barriers are reaction processes that occur in mosquitoes in response to pathogens. Depending on the site of infection and the state of infection, different responses occur in mosquitoes with the goal of eliminating the pathogen. In the case of pathogens, the goal is to successfully establish infection in the host.

### Midgut

The midgut consists of a narrow anterior end and a wider posterior end, which are involved in the absorption of sugar and blood, respectively [7]. After the absorbed blood reaches the midgut, drastic changes in the epithelial cells of the midgut can be observed, such as smaller nuclei, enlarged mitochondria, the disappearance of vesicles of the rough endoplasmic reticulum and the appearance of vortices, an increase in the number of residual lysosomes, and the accumulation of charged opaque substances between the cells [8]. The peritrophic membrane (PM, assembled from chitin and proteins) is secreted by the epithelium into the midgut to protect the epithelial cells from damage; it compartmentalises the midgut and therefore the blood meal digestion and regulates the passage of molecules [9, 10]. So, it could act as a protective barrier against pathogens. Pathogen proliferation in the midgut is greatly restricted by mosquitoes; for example, ookinetes can be lost at a rate of 0.05–1.00 × 10^4^-fold in the midgut of different mosquito species [11], and infection of the midgut triggers the production of large amounts of AMPs [12] and immune proteins in other tissues.

The current study demonstrates the presence of MIB and MEB in the midgut. In refractory mosquitoes with MIBs, the pathogen is unable to infect and replicate in mosquito midgut cells. There are several hypotheses that explain the mechanisms leading to MIB, including the transfer of pathogens to chitin-lined sacs in mosquitoes; compartmentalisation of pathogens (e.g. viruses) by the PM [13]; digestion of pathogens by enzymes in the midgut leading to inactivation; pathogen-midgut interactions that prevent binding [14]; lack of surface receptors in the midgut epithelium [9]. In the presence of MEBs, the pathogen can replicate in the midgut, even to high titres, but the virus may not be able to leave the midgut and spread the infection. Factors such as pathogen load or the dose and duration of pathogen transport determine whether pathogens successfully escape from the midgut [15]. The following two main hypotheses explain the MEB: the direct use of basal lamina and tracheal cells as a conduit between the midgut and the haematopoietic sheath [15]. MEB is dose-dependent [16, 17], and MEB also plays an important role the innate immune memory of pathogens in mosquitos [18].

### Haemocoel

The haemocoel plays an extremely important role in the innate immunity of mosquitoes as pathogens penetrate the midgut and then enter the haemocoel, where they reach the rest of the body through the autodriven or the haemolymph circulation; this is the site where most cellular and humoral immunity occurs [19]. Once the pathogen enters the haemocoel, it is in an environment rich in immune cells and humoral immune factors consisting of haemocytes, pericardial cells, and fat bodies. Of these, haemocytes are the main immune cells in the mosquito. The mechanism of action in the antiviral immunity of haemocytes is unclear and direct evidence is lacking [20]. However, some studies have shown that arboviruses are tropic for mosquito haemocytes [21–23]. In addition, haemocytes are immune surveillance cells that initiate the immune response and have a variety of functions that may contribute to antiviral immunity, including the production of PRRs and proteins responsible for phagocytosis and nodulation, as well as other molecules (e.g. melanisation modulators and enzymes, signal transduction proteins, stress-responsive proteins, and AMPs) to kill or isolate pathogens [24].

Haemocytes are mainly classified into prohaemocytes, oenocytoids, and granulocytes, which exist as both circulating (within the haemolymph) and sessile (in the tissues) [1, 25, 26]. Granulocytes make up 80%–95% of circulating haemocytes and are phagocytic [27]. Oenocytoids make up about 10% and are the main producers of PO [28]. However, other studies have shown that haemocytes may produce a variety of factors involved in melanisation, including POs, serine proteases (SP), serine protease inhibitors (SRPN), phenylalanine hydroxylase, dopachrome convertase and C-type lectins (CTLs), as well as cytotoxic factors such as ROS and RNS [24, 29–32]. In addition, multiple haemocytes aggregate and bind to bacteria to form multicellular sheaths, called encapsulation or nodulation, which is the primary defence response for the removal bacteria from the haemolymph [33, 34].

### Salivary glands

The salivary glands play a major role in disease transmission, and invasion of the salivary gland epithelium and migration to the salivary gland ducts is necessary for most pathogens to complete their life cycle. Salivary glands secrete a variety of proteins involved in different activities, such as CTLs that bind to specific carbohydrates on the surface of microorganisms and are the primary PRRs [35–38]; adenosine triphosphate bisphosphatase (apyrase), which is involved in blood feeding and helps mosquitoes locate blood [39]; and D7, which interferes with haemostasis and vertebrate immune responses [40]. To date, little is known about the role of the salivary glands as immunologically active organs in the anti-pathogen response, but saliva contains complex protein-peptide mixtures, antimicrobial agents, anticoagulants, proteins with angiogenic or anti-inflammatory properties, and immunomodulators, which can act in conjunction with pathogens that infect the host [41].

Furthermore, the molecular mechanisms underlying the SGIB and the SGEB in the salivary glands have not been clearly defined [42]. The concepts of the SGIB and SGEB are tentatively supported by different pathogen infection experiments in different mosquito species, including La Crosse virus (LACV) in *Aedes triseriatus*, Eastern equine encephalitis virus (EEEV) in *Ae. albopictus*, and Japanese encephalitis virus (JEV) and West Nile virus (WNV) in *Ae. aegypti* [43–45]. The SGIB has been demonstrated in *Culex annularis* infected with Western equine encephalitis virus (WEEV) and EEEV [16]. These viruses spread from the midgut to the fat body but cannot enter the salivary glands. Experiments with Rift Valley fever virus (RVFV) infection in the genus *Aedes* provide evidence that the basal lamina surrounding the salivary glands acts as a major barrier to infection [46]. Another study described the role of haemolymph in the SGIB, with the haemolymph of *Culex tarsalis* being more susceptible to WEEV infection compared to refractory females [14]. According to the study, the SGEB appeared in *Aedes* and *Culex* mosquitoes transmitting LACV, SINV, and RVFV [47–50]. The study likewise found that AgESP contributes to the passage of *Plasmodium* through the SGEB of *Anopheles gambiae* and that *Plasmodium* successfully completes its life cycle in mosquitoes by modifying the actin cytoskeleton of mosquito epithelial cells, a process that AgESP may be involved in regulating [17].

### Fat body

The fat body is a major metabolic organ and a primary source of haemolymph proteins [51]; it participates in energy metabolism and reproduction by providing precursors for flight and yolk protein synthesis [52] and plays an important role in the innate immune response. The expansion of the fat body from the body wall to the visceral organs is hypothesised to increase the surface area of the organs and enhance communication with the haemocoel [53]. The fat body consists primarily of trophocytes and oenocytes but is generally thought to include sessile haemocytes, pericardial cells, peripheral neurons, and associated tracheoles [54, 55]. Trophocytes are mesodermal cells that provide energy and nutrients for locomotion and reproduction [56]. Oenocytes are ectodermal cells whose primary functions are detoxification, lipid metabolism, and, to a lesser extent, the maintenance of homeostasis in the mosquito; they are immunocompetent. Transcriptomic analysis revealed that oenocytes express many immune-related genes, with the most abundant transcript being lysozyme P. In addition, the peripheral position of oenocytes may contribute to the recognition of pathogens to activate the innate immune system, which in turn elicits the expression and secretion of immunity factor [57].

In insects, the fat body is the main organ that responds to microbial invasion and is able to secrete AMPs that can rapidly reach effective concentrations against invading microorganisms [58]. In mosquitoes, the fat body is equally involved in several immune pathways and AMPs synthesis in response to bacterial and *Plasmodium* infections [59]. Some of the fat body transcripts are thought to encode immune-related proteins, including defensins (DEFs), cecropins (CECs), CTLs, gram-negative binding proteins (GNBPs), and peptidoglycan recognition proteins (PGRPs) [52, 60]. In addition, enhancement of insulin signalling within the fat body of *Anopheles stephensi* enhances the host immune response to bacterial and *Plasmodium falciparum* infections [61]. Several studies have shown that altering the immune function of mosquito fat body through transgenic techniques can make mosquitoes susceptible to pathogens or develop resistance [62]. It has also been found that proliferation of oenocytes expressing DBLOX peroxidase maintains immune priming in mosquitoes [63].

## Pattern recognition receptors (PRRs) and pathogen-associated molecular patterns (PAMPs)

The activation of the mosquito innate immune system first starts with the recognition of pathogens. When a pathogen infects a mosquito, it is recognised by the host through molecular interactions between PRRs and PAMPs. A genomic analysis of *A. gambiae* revealed the presence of nearly 150 PRRs, the vast majority of which clustered as members of several gene families. Usually, PRRs are host-secreted proteins found in different body parts, such as the midgut, and have an adhesive structural domain that interacts with PAMPs.

### Thioester-containing proteins (TEPs)

TEPs are normally found in haemolymph and are primarily involved in bacterial and *Plasmodium* infections. Their role as essential pathogen recognition molecules in *Drosophila melanogaster*, *A. gambiae*, and *Ae. aegypti* allows for the neutralisation of pathogens [64–66]. In mosquitoes, studies on TEP have focused on TEP1 produced by haemocytes, which is phagocytic and inactive on its own and requires activation by proteolytic cleavage [67]; TEP1 then stabilises itself by forming a complex with the leucine-rich repeat (LRR) structural domains of the LRIM1 and APL1C proteins [3]. Only after the formation of the complex can stable TEP1 bind to bacteria in the haemolymph and *Plasmodium* in the midgut, thus leading to clearance [68–70]. Variants or polymorphisms in the TEP1 and APL1 sequences were associated with the efficiency of *Plasmodium* in killing mosquitoes [71]. The role of TEPs was similarly evaluated in *Ae. aegypti* infected with dengue virus (DENV) and WNV. RNAi-mediated overexpression and silencing of TEP1 and TEP3 revealed that the viral load was reduced when TEP1 was overexpressed, but TEP3 overexpression did not lead to a reduction in viral load, further illustrating the role of TEP1 in regulating viral infection [65].

### Fibrinogen-related proteins or fibrillin (FREPs or FBN)

FREPs are the largest family of pattern recognition receptors in *A. gambiae*, with 59 putative members identified, and 37 FREPs have been identified in *Ae. aegypti*, most of which had upregulated expression in response to bacterial, fungal, or *Plasmodium* infection [10, 72, 73]. Using RNAi-mediated gene silencing, members of the FREP family were found to play a crucial role in the innate immune response and maintenance of immune homeostasis in mosquitoes [74]; furthermore, silencing of fibrinogen-related protein 30 (FBN30) was able to markedly promote parasitic infections, whereas eliminating FBN1 transcripts resulted in the absence of parasites within the mosquito [75]. The most studied member of this family is FBN9, which is associated with antibacterial and *Plasmodium* immunological mechanisms, as FBN9 interacts with bacteria and binds directly to *Plasmodium* in midgut epithelial cells, leading to their destruction [76, 77]. A comparative analysis of FNB9 gene sequences in four neotropical anopheline species (*Anopheles davidianus*, *A. nueces*, *A. salinarius*, and *A. albopictus*) and *A. gambiae* was also carried out [78], and clustering analysis showed that neotropical anophelines and *A. gambiae* belonged to two subgenera in different evolutionary branches.

### C-type lectins (CTLs)

CTLs are membrane-bound or soluble proteins that bind to carbohydrates in a calcium-dependent manner and recognise pathogens by means of cell adhesion and cell-cell interactions, among other interactions. In addition, CTLs promote the activation of prophenoloxidase and melanin deposition on pathogen surfaces in insects [79]. CTLs can induce both positive and negative feedback regulation in the immune response of mosquitoes. In *A. gambiae*, CTLMA2 and CTL4 of this family inhibit melanisation in the midgut, whereas in the haemocoel, they can be present as disulfide-linked heterodimers that kill *Escherichia coli* in a melanisation-independent manner. RNAi-mediated silencing or knockdown of these proteins resulted in increased bacterial loads in the bloodstream and reduced mosquito survival, suggesting that both proteins play a role in the antimicrobial response in mosquitos [38, 80]. In *Ae. albopictus*, the mannose-binding C-type lectin Aalb_CTL is equally involved in combatting yeast and gram-positive bacterial (G+) infections [15]. However, in *Ae. aegypti*, galactose-specific C-type lectin 1 (mosGCTL-1) promotes WNV adhesion to cell membranes, and mosGCTL-3 is the main factor that promotes DENV2 infection [37].

### Gram-negative binding proteins (GNBPs) and peptidoglycan recognition proteins (PGRPs)

GNBPs and PGRPs are the first PRRs to be studied in mosquitoes and can be found in different tissues, such as the midgut and salivary glands; these proteins play an important role in the recognition of bacteria and parasites [81]. In *A. gambiae*, six family members are recognised as PRRs, which act by binding to β-1,3-glucan and lipopolysaccharide on the surface of the pathogen. All members of this family are increased after infection but vary in their antimicrobial specificity and activity. GNBP4 is implicated in the regulation of immune signalling and plays an important role in *E. coli*, *Staphylococcus aureus*, and *Plasmodium berghei* infections but not in *P. falciparum* infections. GNBPA2 is an important factor involved in the killing of *E. coli* and *P. falciparum*, but it has little effect on *P. berghei* and is not involved in *S. aureus* infections [82]. PGRPs recognise and bind peptidoglycan on the surface of pathogens. In *Armigeres subalbatus* infected with *E. coli* and *Micrococcus garciniae*, AsPGRP-S1 expression was increased after infection, and after silencing by RNAi, it was found that AsPGRP-LCs may be involved in mosquito survivability but they are not the main factor in regulating bacterial infection [83].

### Immunoglobulin superfamily (IgSF)

The IgSF specifically recognises and binds to cell surface receptors [6], and the IgSF in *A. gambiae* consists of 138 genes, 85 of which have upregulated expression after infection and are involved in resistance to *Plasmodium* and bacterial infections. One of the infection-responsive genes with an immunoglobulin domain (IRID), IRID4 and IRID6, is involved in killing *P. falciparum conidia*; IRID3 and IRID4 control the growth of opportunistic bacteria, and IRID3, IRID5, and IRID6 are involved in scavenging exogenous bacteria [84]. Another member of this family, AgDSCAM, has been shown to modulate bacterial infection and kill *Plasmodium* in the midgut [77].

## Immune signalling pathways

**Fig. 2:**
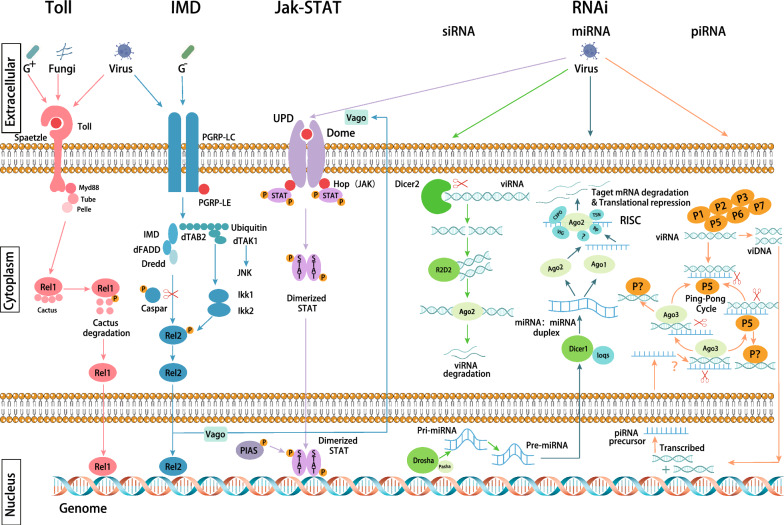
Immune-related signalling pathways in mosquitos. In the Toll pathway, the pathogen first binds to PPRs, which triggers the proteolytic cleavage of the cytokine Späetzle and then activates the Toll receptor to which it binds. Subsequent triggering of signals via the adaptor proteins MyD88, Tube, and Pelle leads to the phosphorylation and degradation of the Cactus protein, an inhibitor of Rel1. Finally, the degradation of the Cactus protein translocates Rel1 to the nucleus and activates the Toll pathway to regulate gene transcription. In the IMD pathway, pathogens bind to PGRP-LC and PGRP-LE ligands and trigger signalling by various cysteoaspartases and kinases. One branch triggers the JNK signalling pathway, while the other triggers signals via IMD, FADD, and Dredd, which mediate phosphorylation, as well as the cleavage of Rel2. Caspar proteins act as negative regulators of the IMD pathway. Subsequent translocation of Rel2 to the nucleus activates the IMD pathway to regulate gene transcription. The JAK-STAT pathway is triggered by the binding of Unpaired (Upd) to the receptor protein Dome, which activates the receptor-associated Hop Janus kinase. Activated Hop kinases phosphorylate each other and subsequently recruit phosphorylated STAT transcription factors and form dimers. Phosphorylated STAT dimers are translocated to the nucleus to activate JAK-STAT-regulated transcription. RNAi pathways include the siRNA pathway, miRNA pathway, and piRNA pathway. In the siRNA pathway, exogenous dsRNA is recognised by the RNA-binding structural domain of Dicer2 and is sheared into small dsRNA of 21–28 nt. The Ago2 protein leads to the silencing of complementary transcripts in the cell by degrading or inhibiting the translation of sRNAs by binding to them, and R2D2 is involved in the delivery of the siRNA guide strand from Dicer2 to Ago2. In the miRNA pathway, pri-miRNAs are produced in the nucleus using the genome and processed by Drosha and Pasha proteins to be converted into pre-miRNAs. Pre-miRNA is translocated to the cytoplasm via export protein 5, where it is further processed by the Dicer1 and Loqs complexes to form miRNA-miRNA duplexes, followed by the formation of guide RNAs via Ago1 and Ago2, leading to degradation of the target mRNAs and transcriptional repression through the RISC complexes (composed of Ago2 or Ago1, VIG, TSN, Rp, C3PO, and other unknown proteins). In the piRNA pathway, after viRNA enters the host cell, it binds to unknown PIWI proteins (PIWI 1–7) and directly enters the ping-pong cycle to bind to the complementary antisense RNA of Piwi5 for shearing and methylation. The antisense RNA is then released to bind to an unknown PIWI protein. The processed positive-sense RNA then binds to Ago3 to form a long-stranded antisense piRNA precursor, and the antisense piRNA precursor is sheared and methylated to form an antisense piRNA release, which binds to Piwi5 to start another round of the ping-pong cycle. The other viRNA undergoes reverse transcription and replication and then enters the host cell nucleus to integrate into the genome, where it is subsequently transcribed and translocated into the cytoplasm to enter the ping-pong cycle

Immune signalling pathways protect mosquitoes from persistent infection by invading pathogens and opportunistic microorganisms and are involved in the regulation of the natural microbiota, such as the gut flora. Immune signalling is an intermediate process that links pathogen recognition and the immune response. Three main immune signalling pathways exist in mosquitoes, namely, the Toll pathway, immune deficiency (IMD) pathway, and Janus kinase (JAK)/signal transducer and activator of transcription (STAT) pathway (Fig. 2). These pathways are activated and trigger immune effector molecules to neutralise invading pathogens, including bacteria, fungi, *Plasmodium*, and viruses (Table 1). In addition, the RNA interfering (RNAi) pathway, although not a classical innate immune signalling pathway, also plays a key role in antiviral defence.

**Table 1:** Studies on mosquito immune signalling pathways

Immune signalling pathways	Mosquito/cell line	Pathogen	Key genes/effectors	References
Toll pathways	*Anopheles gambiae*	*Plasmodium berghei*	Toll, Rel1, Cactus, LRIM1, Tep1	[87]
	*Aedes aegypti*	*Plasmodium gallinaceum*, fungi, DENV, SINV, WNV	RUNX4, AaREL1, Spz1A, Serpin-27A, AMPs, Dif, Späetzle 5	[88–92, 94, 95, 97]
IMD pathways	*Anopheles gambiae*	Bacterium, *Plasmodium berghei*, *P. falciparum*	Caspar, Rel2, AMP, cecropin 1, TEP1, APL1, LRIM1	[98–100]
	*Anopheles stephensi*	*Plasmodium falciparum*	Rel2	[99, 102]
	*Anopheles albimanus*	*Plasmodium falciparum*	Rel2	[99]
	*Aedes aegypti*	DENV, SINV	Caspar, Rel2	[12, 92, 103, 105]
	*Aedes albopictus* U4.4 cell	SFV	–	[104]
JAK-STAT pathways	*Anopheles gambiae*	Bacterium	STAT-A, STAT-B	[106]
	*Aedes aegypti*	DENV	STAT, Dome, Hop, PIAS	[107]
	*Anopheles aquasalis*	*Plasmodium vivax*	STAT, PIAS	[108]
siRNA pathways	*Anopheles gambiae* 4a-2s4 cell	FHV, NoV	Ago2	[116]
	*Aedes aegypti*	YFV, DENV2, SINV	Dicer2, R2D2, Ago2	[117–119]
	*Anopheles gambiae*	ONNV	Ago2	[121]
miRNA pathways	*Anopheles gambiae*	*Plasmodium falciparum*	aga-miR-305	[124]
	*Aedes aegypti*	ZIKV, DENV	–	[125, 126]
	*Aedes aegypti* Aag2 cell	WNV	KUN-miR-1, GATA4	[129]
	*Aedes albopictus* C6/36 cell	DENV, WNV	DENV-vsRNA-5, Aae-miR-2940-5p, aal-miR-4728-5p, miR-1767, miR-276-3p, miR-4448, KUN-miR-1, GATA4	[127–130, 132]
	*Aedes albopictus*	DENV2	miR-252	[131]
piRNA pathways	*Aedes aegypti*	CHIKV	vpiRNA	[137]
	*Aedes albopictus*	CHIKV	vpiRNA	[137]
	*Aedes aegypti* Aag2 cell	SINV, SFV	Piwi5, Ago3, vpiRNA, Piwi4	[135, 139, 141]

### The Toll signalling pathway

The Toll pathway was originally discovered during the screening of genes associated with early embryonic development in *D. melanogaster* and was later found to play a crucial role in defence against fungi, gram-positive bacteria, and viruses [85–87]. The Toll pathway has reshaped the understanding of the immune system not only in *Drosophila* but also in other insects and even mammalian systems. In mosquitoes, genes regulated by the Toll pathway are controlled by the transcription factor Rel1 of nuclear factor kappa B (NF-κB). Silencing Cactus, a negative regulator of Rel1, to induce the Toll pathway significantly reduces the extent of *P. berghei* and *Plasmodium gallinaceum* infection in the midgut of *Anopheles* and *Aedes* mosquitoes. Cosilencing Rel1 and Cactus makes mosquitoes more susceptible, suggesting that Cactus-mediated susceptibility is controlled by Rel1 [88, 89]. Cosilencing of Cactus and LRIM1 or Tep1 also renders mosquitoes susceptible, suggesting that these two effector molecules are induced by the Toll pathway [88].

In addition to the induction of antimalarial activity by Rel1, the Toll pathway is involved in the control of fungal and DENV infections [90, 91]. Knockdown of Rel1 increases mosquito susceptibility to fungus and attenuates the induction of Spaetzle 1A and Serpin-27A [90]. Serpin-27A is overexpressed in heat-killed bacterial Aag2 cells as well as in fungus-infected female *Ae. aegypti* mosquitoes [92]. DENV infection of *Ae. aegypti* activates the transcription of Toll pathway-associated factors and putative effectors (Späetzle, Toll, Rel1A, and multiple AMPs) [91, 93, 94]. The Toll pathway begins to exert antiviral effects as early as 3 days after DENV infection, is able to protect against DENV infection in multiple serotypes, and remains active in different *Ae. aegypti* strains [95]. Furthermore, the symbiotic *Wolbachia* in mosquitoes can inhibit DENV replication by inducing mosquitoes to produce ROS to activate the Toll pathway and subsequently produce AMPs and DEFs [96]. The transcription of Dif, a Toll pathway transcription factor, is induced early in the infection of *Ae. aegypti* with Sindbis virus (SINV) [12]. The role of the Toll pathway in defences against WNV infection is unclear; *Culex quinquefasciatus* infection with WNV does not significantly alter the expression of Toll pathway-associated genes or effectors [97], whereas *Ae. aegypti* infection downregulates the expression of mosquito homologues of *Drosophila* Späetzle 5 [98]. In addition, it has been found that activation of Toll signalling in *A. gambiae* mosquitoes by silencing Cactus modulates haemocyte differentiation leading to a large increase in circulating megacytes (from 2% to 80% of granulocytes) [99]. Moreover, megacytes are recruited to the midgut during *Plasmodium* infection and release microvesicles that promote the activation of the mosquito complement-like system, thereby eliminating *Plasmodium* ookinetes and enhancing mosquito immunity.

### Imd signalling pathway

The Imd pathway likewise plays a crucial role in mosquito immunity. The molecules acting in this pathway overlap with those in the Toll pathway; together, they trigger an immune response. Similar to Rel1 in the Toll pathway, Rel2, which belongs to the same family, plays a central role in the Imd signalling pathway and is involved in the regulation of major AMPs and cecropin 1. The Imd pathway not only plays an important role in antimicrobial defence but also directs the immune response to *Plasmodium* [100–103]. This pathway also has an indirect effect on the viral load of *Ae. aegypti* [104]. Knockdown experiments showed that two isoforms of Rel2 (Rel2-F and Rel2-S) in *Anopheles* mosquitoes are involved in immune defence against gram-positive and -negative bacteria, respectively. Rel2-F is also involved in regulating the intensity of *P. berghei* infection [100]. Although both the Toll and Imd pathways are involved in the immune response to *P. berghei*, the immune response to *P. falciparum* is mainly controlled through the Imd pathway. Caspar proteins are negative regulators of Rel2, similar to the function of Cactus for Rel1. Several studies have shown that silencing Caspar may enhance the immune response to *Plasmodium* [102]. In addition, overexpression of Rel2 rendered *A. gambiae*, *A. stephensi*, and *Anopheles albimanus* completely resistant to *P. falciparum* [101].

Related studies have shown that some Imd pathway-associated proteins and effectors can promote DENV and SINV infection [12, 93]. However, the transient activation of the Imd pathway through RNAi-mediated Caspar silencing did not affect the DENV titre in the midgut of mosquitoes [91]. Furthermore, in experiments where SFV infected *Ae. albopictus* cells, the addition of heat-inactivated gram-negative bacteria (activating both the Imd and JAK-STAT pathways) prior to infection resulted in a reduction in SFV load [105]. Another study showed that disruption of the Imd pathway in DENV-tolerant strains of *Ae. aegypti* resulted in a decrease in midgut DENV titres [106], but DENV titres were unchanged in susceptible strains, suggesting that the Imd pathway may be needed for defence against DENV but that in susceptible strains the pathway may already be operating at maximum load.

### JAK/STAT signalling pathway

The JAK/STAT pathway, a major signalling pathway induced by interferon, is one of the less studied immune pathways in mosquitoes. In *A. gambiae*, this pathway is regulated by two transcription factors, STAT-A and STAT-B. STAT-B regulates the transcription of STAT-A, and bacterial infection leads to the translocation of STAT to the nucleus [107]. Whereas only one type of STAT is present in *Ae. aegypti*, the JAK/STAT pathway plays an important role in the immune response against DENV infection in *Ae. aegypti*. DENV replication in the mosquito midgut was significantly increased when the pathway was inhibited by RNAi-mediated silencing of transmembrane proteins (Dome receptors) or JAK immediate homologues (Hop proteins). In contrast, viral replication was inhibited when an inhibitor (negative regulator PIAS) of activated STAT in the pathway was silenced [108]. Another study showed that the JAK/STAT pathway is involved in the immune response against *Plasmodium intermedius* infection in *A. aquasalis* [109] and found that the transcript levels of STAT and PIAS were elevated 24 and 36 h after *Plasmodium* infection. In addition, in uninfected *Plasmodium* mosquitoes, STAT and PIAS are expressed mainly in the fat body. After infection, they are expressed in other tissues, and silencing STAT increases the number of oocysts in the midgut.

### RNAi pathway

RNAi is a highly conserved, sequence-specific mechanism of gene silencing at the posttranscription level that is primarily involved in the antiviral immune response of mosquitoes. RNAi is a process by which double-stranded RNA (dsRNA) induces small RNA (sRNA) molecules and thus efficiently and specifically degrades homologous mRNA. The RNAi pathway mainly produces sRNA molecules with different characteristics; endogenous small interfering RNAs (siRNAs, 18–24 nt), microRNAs (miRNAs, 18–24 nt), and PIWI protein-interacting RNAs (piRNAs, 24–30 nt) are produced, with siRNAs being the main molecules in the antiviral response [26, 110, 111]. In addition to the small molecules mentioned above, exogenous siRNAs of viral origin may also trigger antiviral responses. These sRNAs form the RNA-induced silencing complex (RISC) by binding to protein molecules such as AGO, TRBP, and PACT, which recognise the target RNA through a specific family of Argonaute (Ago) proteins, leading to gene silencing.

#### siRNA

The siRNA pathway is mainly triggered by Dicer2, R2D2, and Ago2 and can be classified as exogenous and endogenous. Exogenous siRNAs are involved in antiviral immunity by processing exogenous RNAs derived from viruses (or viRNAs), and endogenous siRNAs are involved in regulating cellular processes by binding endogenously encoded dsRNAs [112]. Exogenous dsRNA is recognised by the RNA-binding structural domain of Dicer, which has ribonucleic acid endonuclease activity (RNase III) and is sheared into small dsRNAs of 21–28 nt. Biosynthesis of endogenous siRNAs primarily involves RNA polymerase IV and RNA polymerase V [113]. Dicer is a key element in the biosynthesis of most sRNA molecules, and its main role is to shear dsRNA into sRNAs [114]. The Ago family plays a central role in the RNAi pathway by binding to sRNAs and thereby degrading or inhibiting their translation, leading to silencing of the complementary transcripts in cells. R2D2 is involved in the delivery of the siRNA guide strand from Dicer2 to Ago2; thereby, R2D2 acts as a bridge to the RNAi pathway [115]. The antiviral effect of siRNA was first demonstrated in *A. gambiae* cells, and Ago2 was found to be involved in inhibiting viral replication [116]. Since then, several studies have found that the siRNA pathway inhibits the replication of yellow fever virus (YFV) [117], DENV2 [118] and SINV [119, 120], and the level of viral replication is significantly increased upon the knockdown or silencing of siRNA pathway-related genes [121].

#### miRNA

miRNAs are small endogenous noncoding RNAs that regulate gene expression by degrading mRNAs or terminating transcription by binding to the noncoding regions at the 3′ ends [122]. The mechanism of miRNAs is similar to that of siRNAs, with the main difference being their location in the cell and the effector proteins involved [123]. siRNAs occur mainly in the cytoplasm, whereas the formation of miRNA precursors (pre-miRNAs) is performed in the nucleus, and they are later transported to the cytoplasm for action. miRNAs are involved in a variety of cellular functions, ranging from development to participation in anti-infection immune mechanisms. miRNAs regulate mosquito immunity mainly by controlling the expression of immune-related genes and genes subject to posttranslational modifications or by directly binding to the genome of pathogens. Studies have shown that aga-miR-305 increases the susceptibility of *A. gambiae* to *P. falciparum*, which replicates itself by utilising miRNAs from reservoirs in host cells [124]. Infection with mosquito-borne viruses such as ZIKV [125], DENV [126, 127], and WNV can similarly trigger miRNA responses, leading to the differential expression of multiple miRNAs. Among them, the study confirmed that downregulation of Aae-miR-2940-5p expression inhibited WNV replication [128]; KUN-miR-1 promoted WNV proliferation in *Ae. aegypti* and *Ae. albopictus* cells by upregulating the expression of the transcription factor GATA4 [129]; aal-miR-4728-5p enhanced DENV2 replication after overexpression in *Ae. albopictus* C6/36 cells [130]; miR-252 inhibited DENV2 infection in *Ae. albopictus* by regulating E protein expression [131]. miR-1767 and miR-276-3p enhanced DENV2 replication in C6/36 cells, whereas miR-4448 inhibited DENV2 replication [132].

#### piRNA

The piRNA pathway is poorly understood, but it has regulatory roles in germline maintenance and development [133], the protection of transposons [134], and the regulation of viral infections [135]. Unlike siRNAs and miRNAs, piRNAs are dependent on PIWI subfamily proteins, mainly Piwi, Aub, and Ago3, which together form the piRNA-induced silencing complex (piRISC) [136]. piRNAs are mainly derived from repetitive sequence elements or piRNA clusters, but viral genomes are another source of piRNAs (vpiRNAs) [137, 138]. vpiRNA was detected in *Ae. aegypti* and *Ae. albopictus* infected with chikungunya virus (CHIKV) [137] as well as in SINV-infected *Ae. aegypti* cells [135]. In siRNA-deficient mosquito cells, cellular lesions were found to be aggravated by the inhibition of vpiRNA expression, suggesting that piRNAs exert their antiviral effects in the presence of defects in the siRNA pathway, indicating that piRNA is complementary to the RNAi pathway in the immune response [137]. In addition, another study showed that in *Ae. aegypti*, a noncanonical PIWI protein involved in the antiviral response, Piwi4 knockdown resulted in increased SFV replication in *Ae. aegypti* Aag2 cells [139]. piRNAs are also involved in host resistance to specific viruses. Endogenous bornavirus-like nucleoprotein elements were found to be reverse transcribed and integrated into primate and rodent genomes, resulting in resistance to bornavirus [140]. In addition, the piRNA pathway may play a role in preventing the transmission of vertically transmitted arboviruses through germ cells [141].

## Immune effects

### AMPs

AMPs are small molecule proteins with in vitro antimicrobial activity that are mainly produced by the Toll and IMD signalling pathways. These two pathways regulate the expression of AMPs by activating different transcription factors, which are increased in the fat body when mosquitoes are infected with pathogens [91, 142]. DEFs, CECs, and gambicins (GAMs) are the three main AMP families in mosquitoes. CECs and GAMs primarily target gram-positive bacteria, and DEF primarily targets gram-negative bacteria. DEF has antimicrobial activity in vitro, but its role as an essential component of the mosquito immune response is still unclear. In *A. gambiae*, knockdown of DEF reduced the survival of *S. aureus*-infected mosquitoes but the mosquitoes did not show resistance to malaria [143]. Sequence analysis of *Ae. aegypti* examining 17 AMPs found that the mRNA expression of 7 AMPs was significantly increased after DENV2 infection [144]. In addition, there was no effect on the survival of bacteria-infected mosquitoes after silencing DEF in *Ae. aegypti* [145, 146]. DEF and CEC inhibit DENV replication in *Ae. aegypti* infected with *Wolbachia* [96]. The mechanism of the antimicrobial response to CECs is not known, but ectopic expression of CECs can limit *P. berghei* infection in *A. gambiae* [147]. *Escherichia coli* and *Streptococcus mucin* also stimulate CEC-A, D, E, F, and N expression in Aag2 cells. GAMs exhibited antimalarial activity in the midgut and antimicrobial activity in the blood lumen, and the silencing of GAMs resulted in increased *P. berghei* loads in *A. gambiae* [148]. Furthermore, the silencing of key factors of the JAK-STAT pathway in Aag2 cells leads to reduced GAM activity against bacterial infections, suggesting that immune signalling plays a central role in AMP production [149].

### NO and ROS

NO and ROS are two important effector components of the mosquito immune response. NO is a multifunctional free radical that is produced during the oxidation of l-arginine to l-guanine and catalysed by nitric oxide synthase (NOS). In the midgut, *Plasmodium* glycosylphosphatidylinositol and *Plasmodium*-derived haemagglutinin obtained through infection of the blood meal induce NOS transcription via the STAT pathway, and the resulting NO kills ookinetes by cleavage. NOS is upregulated in the haemocoel after *E. coli* and *Micrococcus luteus* infections, and NO is necessary for bacterial killing and mosquito survival during *E. coli* infection [150]. ROS are involved in the clearance of ookinetes from the midgut and bacteria from the haemocoel, and although the exact mechanism of action on bacteria is unknown, ROS kill *Plasmodium* via the cleavage and melanisation pathways [151–153]. Oral antioxidants also reduced melanisation [151]. The L3-5 strain of *A. gambiae* (*P. berghei* resistant) lives in a state of chronic oxidative stress that promotes the melanisation of ookinetes in the midgut epithelium [152]. In contrast, G3-susceptible strains kill ookinetes through an infection-induced oxidative stress cleavage mechanism that can be maintained by the inhibition of catalase [153]. The silencing of catalase promotes *Plasmodium* infection in the midgut and reduces the bacterial load. In addition, silencing of the dioxygenase DuoX reduced ROS levels and promoted the proliferation of gut microbes [154].

### Melanisation

Melanisation is another important immune response developed by mosquitoes against invading pathogens and is a complex mechanism. The major components involved in melanisation include PO, SP, SRPN, and serine protease hairpin domains (CLIPs). Melanisation initiates the sequential cascade of SPs via PAMPs, which catalyse the hydrolysis of phenol oxidase proteins (proPOs) to active PO. PO is a key enzyme in melanisation, where tyrosine is hydroxylated to DOPA by PO; DOPA is further oxidised to dopaquinone, and in the presence of thiols, dopaquinone forms a cysteine and glutathione coupling to form yellow/red pheomelanin, or in the absence of thiols, dopaquinone is spontaneously converted to dopachrome, which is further converted to eumelanin in brown/black polymers [155], which encapsulate and destroy invading pathogens. SPs and POs are involved in melanisation, and SRPN acts as an inhibitor of serine protease to tightly control the SP cascade [156]. SRPN regulates the activation of proPOs and may activate the Toll pathway to cause *Plasmodium* lysis [157]. At least three SRPNs were found to be involved in the anti-*Plasmodium* innate immune response in *A. gambiae*, with Ag-SRPN6 primarily inhibiting *Plasmodium* proliferation and transmission [158]. CLIPs are similarly involved in regulating the melanisation of *Plasmodium*. SPCLIP1, an arthropod-specific noncatalytic serine protease, is involved in TEP1 accumulation on the surface of invading pathogens to regulate the complement pathway in mosquitoes, thereby inducing a peak in the *Plasmodium* and bacterial melanisation cascade [159]. Another study found that SRPN7 and CLIP2 play a synergistic role in the cascade signalling of mosquitoes during *Plasmodium* infection, with SRPN7 acting as an inhibitor and CLIP2 acting as a facilitator [160]. However, neither SRPN7 nor CLIP2 were transcriptionally regulated following infection with *P. berghei*. CTLs also inhibit the melanisation of mosquitoes against *P. berghei*, and the silencing of CTL4 and CTLMA2 causes LRIM1-dependent ookinete melanisation [80]. The silencing of the key regulators of melanisation TEP1 and CLIPA8 completely prevented mycelial melanisation but not melanisation in conidia and shoot tubes and resulted in more rapid proliferation of the fungus and greater susceptibility to *Coccidioides albicans*. In addition, amino acid metabolism has an important role in melanisation, and tyrosine synthesis is reduced following the silencing of phenylalanine carboxylase (PAH) of the amino acid metabolic pathway, thus altering melanisation in response to *P. berghei*. Carbidopa, an inhibitor of DOPA decarboxylase, similarly affects egg melanisation [161]. Intermediates produced during hydrolysis of ProPOs inactivate SFV; conversely, SFV can activate the melanisation cascade of POs to inhibit virus transmission [162].

### Apoptosis

Apoptosis leads to programmed cell death (PCD) and is essential for maintaining homeostasis because it removes damaged and infected cells. Apoptosis inhibits viral replication as an intrinsic response to viral infection in vertebrates [163, 164]. Although pathogen infection in insects often leads to non-pathogenic infections, there is growing evidence that apoptosis has an antiviral effects in insects [165]. Apoptosis in mosquitoes was found to be involved in the regulation of pathogen load. An apoptosis inhibitor antagonist gene, michelob-x (mx), was first identified in *A. gambiae*, which is similar to the reaper-like apoptosis inhibitor antagonist gene in *Drosophila* and may be involved in regulating the pro-apoptotic response to viral infection [166]. *Aedes aegypti* (CuniNPV-refractory) larvae infected with CuniNPV rapidly generated mx in the midgut and induced the rapid apoptosis of infected cells at 4–6 h, whereas rapidly induced apoptosis was not detected in the larvae of *Cx. quinquefasciatus* (CuniNPV-susceptible). This finding suggests that apoptosis plays an important role in mosquito resistance to viral infection [167]. This phenomenon has been demonstrated in WNV-infected *Cx. quinquefasciatus* [168, 169] and DENV-infected resistant and susceptible *Ae. aegypti* [165]. There are two important factors in the mosquito apoptosis pathway, Aeiap1 and Aedronc. Aedronc is an RNAi-mediated primary apoptotic cysteinyl asparaginase, and the silencing of Aedronc was found to increase the prevalence of infection in resistant *Ae. aegypti* mosquitoes [170]. Aeiap1, an inhibitor of apoptosis, activates apoptosis by silencing IAP, leading to increased SINV titres in the midgut and viral spread to other sites in *Ae. aegypti*; meanwhile, silencing Aedronc has the opposite result [171]. These results are contrary to the hypothesis that apoptosis plays a role in the mosquito antiviral immune response. Apoptosis may limit viral infectivity by disrupting the physical barriers of the cell, but when apoptosis is artificially induced, both viral infectivity and transmission are enhanced.

### Autophagy

Autophagy is a fundamental cellular process involved in the maintenance of cellular homeostasis in situations of stress and nutrient deprivation. Autophagy can activate or modulate the immune response and directly eliminate intracellular microorganisms [172]. Autophagy-related gene 5 (ATG5) in the midgut of DENV2-susceptible *Ae. aegypti* was transcriptionally elevated upon infection with DENV2, in contrast to resistant strains, and this elevation was associated with increased expression of apoptosis-related genes [173]. When these genes were silenced, a concomitant increase in DENV2 load was accompanied by accumulation of Atg8-PE, a marker of autophagy induction and progression [173]; furthermore, apoptosis-related genes are also involved in regulating autophagy.

### Phagocytosis

Phagocytosis is a conservative immune process that has evolved to effectively neutralise or remove microorganisms. By recognizing that particles or microorganisms are excited and form phagosomes after being engulfed by phagocytes, the phagosome fuses with the lysosome, and the microorganism is digested by hydrolytic enzymes within the lysosome. In mosquitoes, the granulocyte subpopulation of haemocytes isolates and kills bacteria by phagocytosis [174, 175], approximately 95% of circulating haemocytes are phagocytic, and a single haemocyte can phagocytose > 1000 bacteria 24 h post infection [176]. The main phagocytosis regulators in mosquitoes are PRRs, transmembrane receptors, and intracellular signalling proteins. TEP1, TEP3, and TEP4 are involved in phagocytosis of gram-positive and -negative bacteria in the haemocoel [67, 177]. TEP1 downregulates bacterial prevalence through thioester-mediated binding, which in turn initiates phagocytosis, and the mechanism of action of TEP3 and TEP4 is unknown [177]. In addition, LRIM1 is also involved in phagocytosis, and its exact mechanism has not been elucidated, but it has been shown that the antimicrobial activity of TEP1 in the midgut depends on the complexation of TEP1 with LRIM1 in the haemocoel [68]. Knockdown of AgDSCAM, a highly variable immunoglobulin structural domain containing the receptor for *A. gambiae*, promoted bacterial proliferation in the haemocoel and reduced mosquito survival [77]; in addition, AgDSCAM recognises bacteria to initiate phagocytosis. Transmembrane receptors trigger phagocytosis by directly recognising pathogens or pathogens regulated by haemocyte proteins, including PGRPs, integrins (BINT2), and low-density lipoprotein receptor-related protein (LRP1) [177, 178]. Intracellular CED proteins can similarly trigger bacterial internalisation. In *A. gambiae*, knockdown of CED2, CED5, or CED6 reduced phagocytic efficiency to 80% [177]. Analysis showed that TEP1-, TEP3-, LRIM1-, and LRP1-mediated phagocytosis occurs through the CED6 pathway, and TEP4- and BINT2-mediated phagocytosis occurs through the CED2/CED5 pathway. Another study on *A. gambiae* found that cytoplasmic actin can act as a pathogen recognition factor by interacting with specific extracellular immune factors, which in turn bind to the bacterial surface to mediate phagocytosis [179].

## Immune priming

In recent years, researchers have discovered that the mosquito's innate immune response can induce an enhanced immunity when it encounters reinfection, a phenomenon known as "immune priming" (also known as trained immunity). The innate immune memory of *A. gambiae* for *Plasmodium* infection is associated with haemocyte differentiation [18], mediated by the systemic release of haemocyte differentiation factor (HDF). *Plasmodium* infection mediates immune initiation in *A. gambiae* by inducing the release of lipoxin/lipocalin complex, which is associated with a sustained increase in the expression of a lipocalin lipid carrier called Evokin, which promotes haematopoietic differentiation [180]. Another study similarly found that prostaglandin E2 (PGE2) released from the midgut of *A. gambiae* attracts haemocytes to the midgut surface and enhances their patrolling activity, resulting in a more effective immune response to re-infection in *A. gambiae* [181]. Double peroxidase (DBLOX) is essential for HDF synthesis, and histone acetyltransferase AgTip60 is essential for oenocyte number, HDF synthesis, and immune priming [182].

It has been previously shown that the response of *Ae. aegypti* to bacterial reinfection is predominantly non-specific immune priming [183]. However, another study found that *A. gambiae* fed with *Serratia fonticola S1* and *Enterobacter sp. Ag1* sugar meal had a higher 24-h survival rate after re-infection and that this immune priming was specific, suggesting that the previous bacterial sugar meal enhanced the subsequent antibacterial immunity [184]. Bacterial exposure in the larval stage of *Ae. aegypti* was found to increase the survival of both male and female adult mosquitoes. However, there was sexual immune dimorphism, with higher PO activity in male mosquitoes and higher NO production, higher antibacterial activity, and longer duration in female mosquitoes [185]. Using the *Ae. aegypti* Aag2 cell line as a model, the investigators found that bacterial-induced immune responses inhibited RVFV replication, and that RVFV infection markedly enhanced subsequent bacterial-stimulated immune responses. RVFV infection affected gene expression of PRRs, which may be involved in immune priming [186].

Mosquitoes are also involved in immune priming during interactions with the gut microorganisms, including *Wolbachia* [187, 188]. It is a major way to increase the survival of reinfected mosquitoes and to limit *Plasmodium* invasion. Studies have also shown that immune priming to viruses [189] and *Wolbachia* [190, 191] sometimes enhances immunity but also suppresses the immune response. The effect of transgenerational immune priming (TGIP) on offspring immune response rates is also being further investigated. *Aedes aegypti* can pass on antiviral immune memory to their offspring, and the memory persists for several generations. CHIKV-infected *Ae. aegypti* offspring show lower viral loads when infected, and TGIP is viral RNA-dependent and sequence-specific but RNAi-independent [192]. In female offspring of *Ae. aegypti* infected with DV, transcripts of the siRNA pathway are reduced and immune priming decreases viral load [193].

## Symbiotic bacteria and anti-pathogenic effects

### Influence of gut microorganisms on immunity

**Fig. 3:**
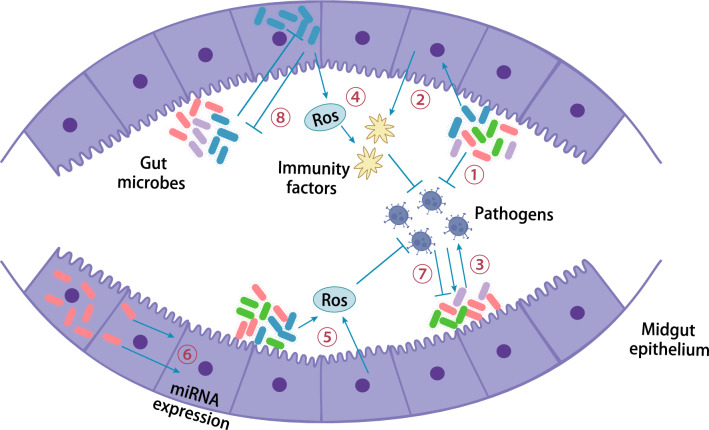
Interactions among mosquitoes, gut microbes, and pathogens. Red-, purple-, green-, and blue-coloured rods with hyphae represent various gut microbes. ① Gut microbes directly inhibit viruses; ② gut microbes can inhibit viruses by stimulating basal immunity; ③ gut microbes can potentiate viruses; ④ gut microbes, such as *Wolbachia*, can stimulate immunity through the production of ROS; ⑤ ROS can also be coproduced by a mosquito host and microbes and inhibit bacteria and pathogens; ⑥ intracellular microbes can modulate the expression of host miRNAs; ⑦ arboviruses can either inhibit or promote gut microbes; ⑧ interactions with gut microbes can affect the microbes themselves

As an important site for pathogen colonisation and determining the outcome of infection, the gut possesses complex microbial populations that influence multiple aspects of mosquito nutrition, reproduction, metabolism, immunity, and vectorial competence. Mosquitoes acquire viruses by sucking blood, which then infects intestinal epithelial cells and spreads throughout the body to various organs and tissues via the haemocoel. Gut microbes play a role in mosquito immune regulation by initiating host immune surveillance mechanisms and secreting metabolites (Fig. 3) [194, 195]. The mosquito innate immune system mediates the interaction of midgut microbes with DENV infection, and *Proteus sp. prsp_P* upregulates AMP expression and increases mosquito resistance to DENV infection [196]. Microbes trigger basal immune and antimicrobial responses against DENV infection, and the immune response triggered by DENV infection also affects midgut microbes. Another study found that in *A. gambiae*, gut microbes reduce infection rates and *Plasmodium* counts by producing antiparasitic effectors. *Chromobacterium Csp_P* in the midgut reduced the survival of larvae and adults and suppressed malaria and DENV infection. Additionally, *Csp_P* mediates in vitro anti-*Plasmodium* and anti-DENV activity by producing bioactive factors with transmission blocking and therapeutic potential [197]. In *Ae. aegypti*, *Talaromyces* alter mosquito physiology by modulating digestive enzymes and trypsin activity to facilitate pathogen infection [198]. *Serratia marcescens* is a key commensal bacterium that plays a role in the efficient acquisition of arboviruses. *Serratia marcescens* facilitates arbovirus infection by secreting the SmEnhancin protein, which digests membrane-bound mucin in the gut epithelium, thereby enhancing virus transmission [199]. The findings suggest that tripartite interactions among mosquitoes, gut microbes, and pathogens offer new possibilities for the control of mosquito-borne infectious diseases.

### *Wolbachia*

Currently, the most widely used microbial control strategy for insect-borne viruses is *Wolbachia*. *Wolbachia* is a maternally inherited intracellular symbiotic bacterium predicted to infect > 60% of insects [200]. *Wolbachia* is programmed to control insects by utilising the following two key processes of host biology: cytoplasmic incompatibility (CI) and pathogen blocking (PB) [201]. CI is a reproductive manipulation that manifests as fewer eggs being produced or the failure to hatch after mating between males infected with *Wolbachia* and uninfected females, the compatibility of females carrying *Wolbachia* with either infected or uninfected males for successful reproduction, and the possibility of CI between mosquitoes carrying different strains of *Wolbachia*, referred to as bidirectional CI. In addition, some *Wolbachia* trans infections also cause PB; *Wolbachia* inhibits pathogen infection, replication, and the transmission of key pathogens. Mosquito control interventions utilising *Wolbachia* generally follow two approaches, namely, population suppression and population substitution [202]. Population suppression targets the two main DENV vectors, *Ae. aegypti* (infection by the wAlbB strain) and *Ae. albopictus* (triple infection by the wAlbA, wAlbB and wPip strains). By releasing large numbers of *Wolbachia*-infected male mosquitoes to mate with wild females, CI then leads to a dramatic decline in the size of the target mosquito population by reducing female fecundity [203–206], with suppression rates of local mosquito populations ranging from 78% to 95%. The *Wolbachia* wMelPop-CLA strain introduced from *D. melanogaster* into *Ae. aegypti* reduced the lifespan of female mosquitoes by approximately 50% compared to uninfected mosquitoes [207]. Population substitution is the use of CI to displace *Wolbachia*-infected populations using mosquito populations that transmit the target virus; therefore, PB limits arbovirus transmission. Studies have shown that population substitution is generally effective and that *Wolbachia* can spread rapidly at high frequencies and remain stable over time in target populations [208–210]. Successful infection with *Wolbachia* usually corresponds to a significant reduction in DEF transmission in DENV-endemic areas [209–212]. In addition, *Wolbachia* increases mosquito resistance to DENV, CHIKV, YFV, and *Plasmodium* infection [213–216]. The *Wolbachia* wAlbB strain from *Ae. albopictus* similarly showed resistance to DENV after midgut colonisation in *Ae. aegypti* [217], and gene expression analysis showed that wAlbB induced ROS production, which in turn induced activation of the Toll pathway [96].

## Conclusions

Mosquitoes, as vectors of many infectious diseases, have received extensive attention worldwide. In recent years, with the development of RNAi and transgenic and CRISPR/Cas9 technologies, there has been an increasing number of studies on mosquito immune mechanisms. This article describes the innate immune mechanisms of mosquitoes in response to pathogens, outlining the physical barriers of the mosquito salivary glands, haemocoel, midgut, and the physiological barriers of MIB, MEB, SGIB, and SGEB, which activate the Toll, Imd, and JAK-STAT immune signalling pathways through the recognition of pathogens by PRRs and PAMPs, thereby triggering cellular and humoral immune responses such as AMPs, melanisation, ROS and NO, apoptosis, autophagy, phagocytosis, and other cellular and humoral immune responses. Finally, the mechanisms and related studies of mosquito immune priming as well as symbiotic bacteria against pathogen infection are described. The study of the molecular mechanisms of mosquito innate immune responses provides new ideas for mosquito density control and the development of mosquito-borne disease control strategies. However, the mechanism of mosquito innate immunity still has many unknowns and needs to be further studied and explored; for example the lacks of knowledge about the molecular and biochemical mechanisms underlying the physiological barriers of MIB, MEB, SGIB, and SGEB; the differentiated mechanisms of innate immunity among mosquito species; the differences in immune mechanisms between larvae and adults; the mechanisms of haemocyte development and differentiation; the complex regulatory mechanisms in response to different pathogens; the exact mechanisms of immune-associated genes and effectors involved in immune regulation; and the search for new mechanisms of immunity, immune genes, and immune effectors all require further investigation. The development of gene editing and genomics and biochemical analysis techniques will further promote the study of mosquito immune mechanisms, which will provide a basis for improving mosquito-borne disease research.

## Data Availability

The datasets supporting the conclusions of this article are included within the article.

## Abbreviations

PRRs: Pattern recognition receptors
PAMPs: Pathogen-associated molecular patterns
AMPs: Antimicrobial peptides
PO: Phenoloxidase
ROS: Reactive oxygen species
NO: Nitric oxide
PM: Peritrophic membrane
MIB: Midgut infection barrier
MEB: Midgut escape barrier
SGIB: Salivary gland infection barrier
SGEB: Salivary gland escape barrier
LACV: La Crosse virus
EEEV: Eastern equine encephalitis virus
JEV: Japanese encephalitis virus
WNV: West Nile virus
WEEV: Western equine encephalitis virus
RVFV: Rift Valley fever virus
TEPs: Thioester-containing proteins
LRR: Leucine-rich repeat
DENV: Dengue virus
FREPs: Fibrinogen-related proteins
FBN: Fibrillin
CTLs: C-type lectins
GNBPs: Gram-negative binding proteins
PGRPs: Peptidoglycan recognition proteins
IgSF: Immunoglobulin superfamily
IRID: Immunoglobulin domain
IMD: Immune deficiency
JAK: Janus kinase
STAT: Signal transducer and activator of transcription
RNAi: RNA interfering
NF-κB: Nuclear factor kappa B
SINV: Sindbis virus
dsRNA: Double-stranded RNA
sRNA: Small RNA
siRNAs: Small interfering RNAs
miRNAs: MicroRNAs
piRNAs: PIWI protein-interacting RNAs
RISC: RNA-induced silencing complex
Ago: Argonaute
YFV: Yellow fever virus
pre-miRNAs: MiRNA precursors
CHIKV: Chikungunya virus
DEFs: Defensins
CECs: Cecropins
GAMs: Gambicins
NOS: Nitric oxide synthase
SP: Serine protease
SRPN: Serine protease inhibitor
CLIPs: Serine protease hairpin domains
proPOs: Phenol oxidase proteins
PCD: Programmed cell death
mx: Michelob-x
ATG5: Autophagy-related gene 5
LRP1: Lipoprotein receptor-related protein
HDF: Haemocyte differentiation factor
PGE2: Prostaglandin E2
DBLOX: Double peroxidase
TGIP: Transgenerational immune priming
CI: Cytoplasmic incompatibility
PB: Pathogen blocking
